# Neuregulin-1 elicits a regulatory immune response following traumatic spinal cord injury

**DOI:** 10.1186/s12974-018-1093-9

**Published:** 2018-02-21

**Authors:** Arsalan Alizadeh, Kallivalappil T. Santhosh, Hardeep Kataria, Abdelilah S. Gounni, Soheila Karimi-Abdolrezaee

**Affiliations:** 1 Regenerative Medicine Program, Department of Physiology and Pathophysiology, Faculty of Medicine, Spinal Cord Research Centre, University of Manitoba, 629-Basic Medical Sciences Building, 745 Bannatyne Avenue, Winnipeg, Manitoba R3E 0J9 Canada; 2 Department of Immunology, University of Manitoba, Winnipeg, Manitoba Canada

**Keywords:** Spinal cord injury, Neuregulin-1, Neuroinflammation, T cells, B cells, Macrophages, Autoantibodies, Cytokines and chemokines, Rat

## Abstract

**Background:**

Spinal cord injury (SCI) triggers a robust neuroinflammatory response that governs secondary injury mechanisms with both degenerative and pro-regenerative effects. Identifying new immunomodulatory therapies to promote the supportive aspect of immune response is critically needed for the treatment of SCI. We previously demonstrated that SCI results in acute and permanent depletion of the neuronally derived Neuregulin-1 (Nrg-1) in the spinal cord. Increasing the dysregulated level of Nrg-1 through acute intrathecal Nrg-1 treatment enhanced endogenous cell replacement and promoted white matter preservation and functional recovery in rat SCI. Moreover, we identified a neuroprotective role for Nrg-1 in moderating the activity of resident astrocytes and microglia following injury. To date, the impact of Nrg-1 on immune response in SCI has not yet been investigated. In this study, we elucidated the effect of systemic Nrg-1 therapy on the recruitment and function of macrophages, T cells, and B cells, three major leukocyte populations involved in neuroinflammatory processes following SCI.

**Methods:**

We utilized a clinically relevant model of moderately severe compressive SCI in female Sprague-Dawley rats. Nrg-1 (2 μg/day) or saline was delivered subcutaneously through osmotic mini-pumps starting 30 min after SCI. We conducted flow cytometry, quantitative real-time PCR, and immunohistochemistry at acute, subacute, and chronic stages of SCI to investigate the effects of Nrg-1 treatment on systemic and spinal cord immune response as well as cytokine, chemokine, and antibody production.

**Results:**

We provide novel evidence that Nrg-1 promotes a pro-regenerative immune response after SCI. Bioavailability of Nrg-1 stimulated a regulatory phenotype in T and B cells and augmented the population of M2 macrophages in the spinal cord and blood during the acute and chronic stages of SCI. Importantly, Nrg-1 fostered a more balanced microenvironment in the injured spinal cord by attenuating antibody deposition and expression of pro-inflammatory cytokines and chemokines while upregulating pro-regenerative mediators.

**Conclusion:**

We provide the first evidence of a significant regulatory role for Nrg-1 in neuroinflammation after SCI. Importantly, the present study establishes the promise of systemic Nrg-1 treatment as a candidate immunotherapy for traumatic SCI and other CNS neuroinflammatory conditions.

**Electronic supplementary material:**

The online version of this article (10.1186/s12974-018-1093-9) contains supplementary material, which is available to authorized users.

## Background

Pathophysiology of spinal cord injury (SCI) is characterized by a robust humoral and cellular neuroinflammatory response that is driven by the interplay between the peripherally recruited leukocytes and resident glial cells [1, 2]. Microglia/macrophages, T cells, and B cells play central roles in orchestrating the innate and adaptive immune responses following SCI through a plethora of inflammatory cytokines, chemokines, antibodies, and proteolytic enzymes [2–4]. Activated microglia/macrophages mediate the initial neuronal injury through pro-inflammatory mediators and oxidative damage [5]. They also activate T cells through their antigen presenting function [1]. Activated T cells stimulate B cells, through a host of cytokines and signaling molecules, to produce autoreactive antibodies against spinal cord tissue [6] causing tissue damage through antibody-mediated cytotoxicity [7, 8]. These processes collectively lead to an imbalanced and dysregulated milieu that impedes repair and regeneration after SCI [1]. Despite their undisputed role in degenerative processes following SCI, emerging evidence indicates that immune cells can be modulated in their microenvironment to adopt regulatory and pro-regenerative phenotypes [9, 10]. Given the profound impact of neuroinflammation on SCI pathophysiology, it is critical to unravel endogenous mechanisms that regulate immune cells after injury. This knowledge is vital for developing immunotherapies that can harness the potential of immune cells in fostering a supportive microenvironment for spinal cord repair and regeneration.

We previously identified that SCI results in a rapid and long-lasting decline in the tissue levels of the neuronally derived growth factor, Neuregulin-1 (Nrg-1) [11]. Nrg-1 is primarily known for its essential role in Schwann cell and oligodendrocyte differentiation, maintenance, and myelination in the central and peripheral nervous systems [12]. In a preclinical model of compressive/contusive SCI in rats, we demonstrated that increasing the deficient bioavailability of Nrg-1 in the injured spinal cord improves neurological recovery following injury [13]. In our efforts to elucidate the mechanisms underpinning the recovery of function after Nrg-1 treatment [13], we identified that Nrg-1 promotes oligodendrogenesis and protects oligodendrocytes and axons resulting in white matter preservation after SCI [11]. Importantly, we found a remarkable positive role for Nrg-1 in regulating astrogliosis and glial scar formation in the injured spinal cord [13]. Moreover, Nrg-1 treatment through intrathecal infusion attenuated the release of pro-inflammatory cytokines, tumor necrosis factor-alpha (TNF-⍺), and interleukin-1 beta (IL-1β) in acute SCI while increasing the tissue levels of the anti-inflammatory cytokine, IL-10 at subacute SCI [13]. Other studies have also shown a neuroprotective role for Nrg-1 in attenuating neuronal injury in vitro and in ischemic brain injury models by reducing neurotoxicity and pro-inflammatory mediators [14, 15]. These initial findings suggest a potential immunomodulatory mechanism of Nrg-1 in fostering a pro-regenerative microenvironment that improves tissue preservation and neurological recovery following SCI [13].

In the present study, we dissected the impact of Nrg-1 on the peripheral and spinal cord immune responses at various stages of SCI. Using a clinically relevant model of severe compressive SCI in rat, we investigated whether systemic delivery of recombinant human Nrg-1 (rhNrg-1) could regulate the recruitment, phenotype, and secretory properties of SCI relevant leukocytes in the blood and injured spinal cord. We demonstrate, for the first time, that Nrg-1 promotes a comprehensive immune regulatory response by macrophages and T and B lymphocytes at acute and chronic stages of SCI through modulation of a repertoire of cytokines and chemokines in the spinal cord tissue. Our new findings establish that bioavailability of Nrg-1 activates a neuroinflammatory process that provides a supportive environment for endogenous repair and recovery of function following SCI.

## Methods

### Rat model of compressive spinal cord injury and animal care

All animal experiments were approved by the University of Manitoba Animal Care Committee in agreement with the Canadian Council on Animal Care guidelines and policies. A total of 120 age and weight matched (8-10 weeks, 250 g) adult female Sprague-Dawley (SD) rats (Central Animal Care Facility at the University of Manitoba) were used in this study. For SCI surgeries, animals underwent laminectomy at thoracic levels T6-T8 under deep isoflurane anesthesia. To induce compressive SCI, a 35-g aneurysm clip (University Health Network, Toronto, Ontario) was applied for 1 min at mid-thoracic (T7) level. Each animal received a mixture of buprenorphine (Temgesic®, 0.05 mg/kg) and meloxicam (Metacam®, Boehringer Ingelheim GmbH, 2 mg/kg) supplemented by three additional doses of buprenorphine every 8 h for pain management. Rats were housed in a 12:12 h light/dark cycle in standard plastic cages before SCI and afterwards in cages covered with soft paper bedding to prevent skin erosions and urine scalding. Pelleted food and drinking water were available ad libitum. Animals received daily examination with their bladders expressed three times a day until regaining full reflexive bladder control.

### Experimental groups and treatments

Prior to surgeries, animals were randomly divided into three experimental groups: (1) uninjured control; (2) SCI/vehicle control, which received vehicle solution used for rhNrg-1β1 delivery (0.1% bovine serum albumin, BSA, in 0.9% saline); and (3) SCI/Nrg-1, which received rhNrg-1β1, 2 μg/day systemically using subcutaneously implanted osmotic mini-pumps (Alzet® model #1003D for 3 days, model #2001 for 7 days, model #2002 for 14 days, and model #2006 for 42 days). The dosage of rhNrg-1β1 (Shenandoah Biotechnology Inc., Cat # 100-46-50) was determined based on our previous study [13] where we found significant structural and functional recovery with a dose range of 0.5 to 1.5 μg per day of rhNrg-1β1 delivered intrathecally into the subarachnoid space. In this study, we delivered Nrg-1 systemically, thus the dose was increased to 2 μg/day to ensure adequate delivery of Nrg-1 to the spinal cord. Treatment was started approximately 30 min after SCI.

### Flow cytometric assessment of spinal cord immune cells

At each end point, animals were deeply anesthetized using a mixture of 40% isoflurane + 60% propylene glycol. Then, animals were sacrificed, and spinal columns were excised and placed on dry ice for 5 min. The spinal cords were then exposed using laminectomy, and 1.5 cm of tissue centering the epicenter of the injury was excised. Mechanical dissociation was performed in Hank’s Balanced Salt Solution (HBSS) using fine scissors, and the tissue was retrieved by centrifugation at 1000 rpm for 1 min at room temperature. Tissue particles were then enzymatically dissociated by incubating with 2.5 mg trypsin + 5 mg collagenase in 5 ml Dulbecco’s modified Eagle’s media (DMEM, 20 min, at 37 °C). Following trituration, the enzymatic reaction was stopped using 10 ml DMEM + 10% fetal bovine serum (FBS) and tissue mixture was filtered through a 40-μm cell strainer. Cells were pelleted and reconstituted in 6 ml of HBSS and overlaid on OptiPrep® (Sigma-Aldrich, D1556) gradient and centrifuged at 1900 rpm for 15 min at 20 °C for separation of myelin debris from immune cells [16]. Supernatant, containing myelin and tissue debris, was carefully discarded, and cells were washed and re-suspended in 2.5 ml of HBSS. Cells were then incubated with RBC lysis buffer (Biolegend, 420301), washed, and counted. A total of 7.5 million cells were harvested from each animal. For each antibody panel, 2 million cells per animal were used. Non-specific binding sites were blocked using 10% normal mouse serum for 30 min (Invitrogen, 10410). Cells were then incubated with antibody cocktail containing surface antibodies for each panel (listed in Table 1) for 30 min in dark at 4 °C. Cells were then fixed using BD™ Cytofix Fixation Buffer for 15 min at 4 °C (BD, 554655).

**Table 1:** List of antibodies used for flow cytometric and immunohistochemical assessment of immune cells

Antibody	Color	Application	Cat number	Concentration
CD3	PerCP	Flow	eBioscience, 46-00-30-82	1:20
CD4	BV510	Flow, IHC	BD, 740138	1:20, (1:100 for IHC)
Ms IgG2a, k	BV510	Flow	BD, 563027	1:20
CD45	APC-Cy7	Flow	BD, 561586	1:20
Ms IgG1, k	APC-Cy7	Flow	BD, 557873	1:20
IFN-γ	FITC	Flow	BD, 559498	1:20
Ms IgG1, k	FITC	Flow	BD, 554679	1:20
IL-10	PE	Flow	BD, 555088	1:20
Ms IgG2b, k	PE	Flow	BD, 555058	1:20
FoxP3	APC	Flow, IHC	eBioscience, 17-5773-80	1:20, (1:100 for IHC)
Ms IgG2a, k	APC	Flow	eBioscience, 17-4724-42	1:20
CD68	FITC	Flow, IHC	Bio-Rad, MCA341F	1:20, (1:100 for IHC)
Ms IgG1	FITC	Flow	Bio-Rad, MCA1209F	1:20
CD163	PE	Flow, IHC	Bio-Rad, MCA342R	1:20, (1:100 for IHC)
Ms IgG1	PE	Flow	Bio-Rad, MCA1209PE	1:20
CD86	BV-421	Flow, IHC	BD, 743,211	1:20, (1:100 for IHC)
Ms IgG1, k	BV-421	Flow	BD, 562438	1:20
IL-10	Alexa 647	Flow	BD, 562156	1:20
Ms IgG2b, k	Alexa 647	Flow	BD, 557903	1:20
CD45RA	APC-Cy7	Flow	BD, 561624	1:20
Armenian hamster IgG isotype control	PE-Cy7	Flow	eBioscience, 25-4888-82	1:20
Anti Iba-1, rabbit	Unconjugated	IHC	Wako, 019-19741	1:500
Anti CD3, rabbit	Unconjugated	IHC	Abcam, GR2952321	1:300
Anti IL-10, mouse	Unconjugated	IHC	R&D, MAB519	1:300
Goat anti-rabbit secondary Ab	Alexa Fluor® 647	IHC	Invitrogen, A21245	1:400
Goat anti-mouse secondary Ab	Alexa Fluor® 568	IHC	Invitrogen, A11031	1:400

**Fig. 1:**
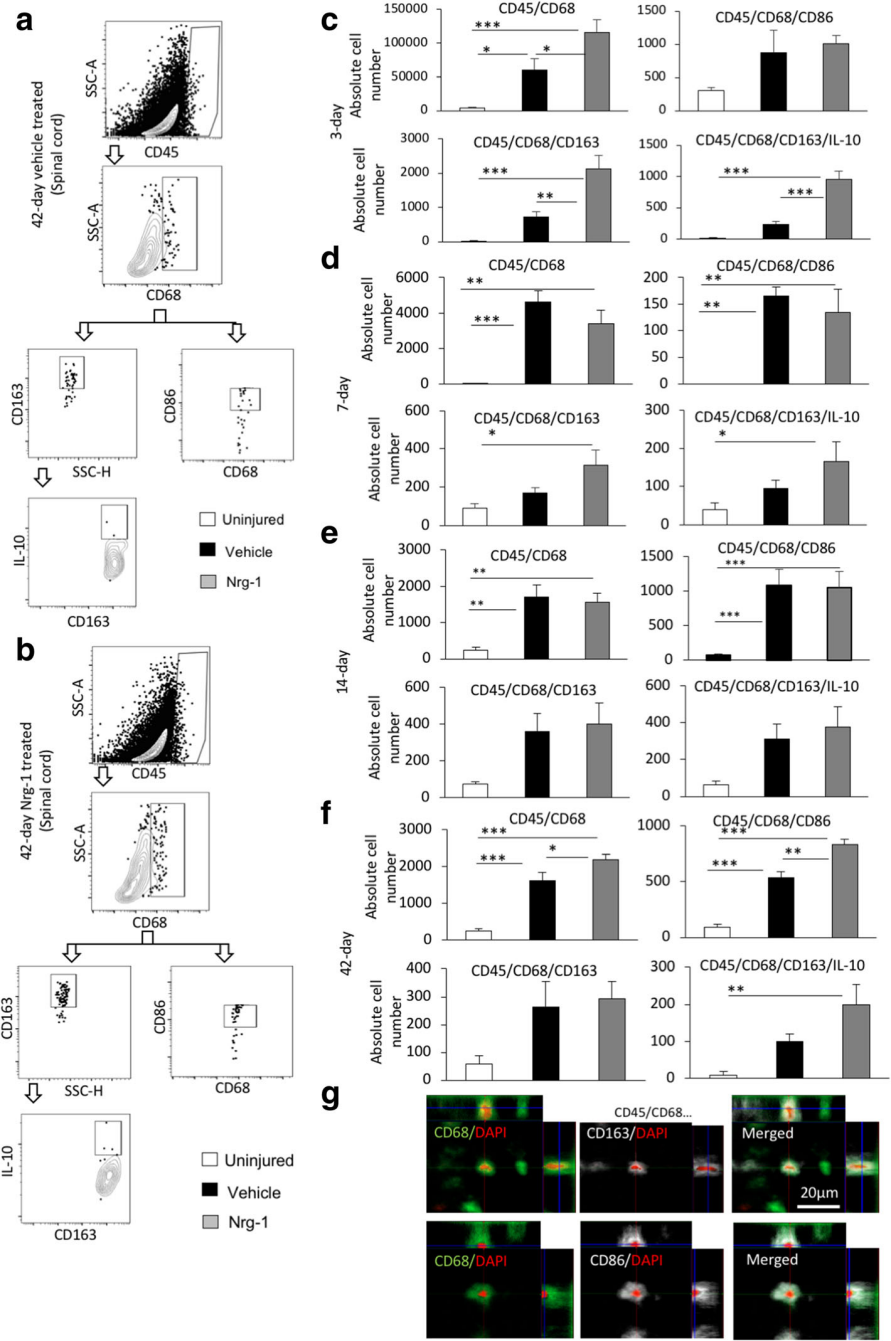
Nrg-1 treatment alters M1 and M2 macrophage populations after SCI. **a**, **b** Representative images of the gating strategy for flow cytometry are provided for infiltrated spinal cord macrophages at 42-day post-injury under each treatment group. **c** Flow cytometric analysis 3 days post-SCI showed a robust increase in the number of macrophages (CD45^+^CD68^+^) in the injured spinal cord. Nrg-1-treated animals demonstrated a significantly higher number of macrophages compared to the vehicle-treated group. Phenotypical analysis of macrophages at 3-day time-point showed no significant difference in the number of infiltrated M1 macrophages (CD45^+^CD68^+^CD86^+^) between vehicle- and Nrg-1-treated groups. However, Nrg-1-treated SCI animals had a significantly higher population of M2 macrophages (CD45^+^CD68^+^CD163^+^ and CD45^+^CD68^+^CD163^+^IL-10^+^) in the spinal cord. **d**, **e** At 7 and 14 days post-SCI, the total number of tissue macrophages and their M1 subpopulation was significantly higher in the injured animals compared to the uninjured group, while M2 macrophage population remained unaltered. There was also no significant difference in the total number of macrophages and their M1 or M2 phenotype between the vehicle- and Nrg-1-treated groups. **f** At 42 days post-SCI, the number of infiltrated macrophages was 30 times less than the acute 3-day time-point in SCI baseline condition but still significantly higher than uninjured animals. Compared to both vehicle and uninjured rats, Nrg-1-treated animals showed a significantly higher number of M1 macrophages. There was no significant difference in the number of M2 macrophages between the vehicle and Nrg-1 treatment groups at this time-point, although Nrg-1-treated rats had a significantly higher number of M2 macrophages as compared to uninjured animals. **g** Immunohistochemical analysis verified the presence of M1 (CD68^+^CD86^+^) and M2 (CD68^+^CD163^+^) macrophages in the perilesional area (*N* = 5/group/time-point, *p < 0.05, **p < 0.01, ***p < 0.001, one-way ANOVA followed by Holm-Sidak post hoc test)

**Fig. 2:**
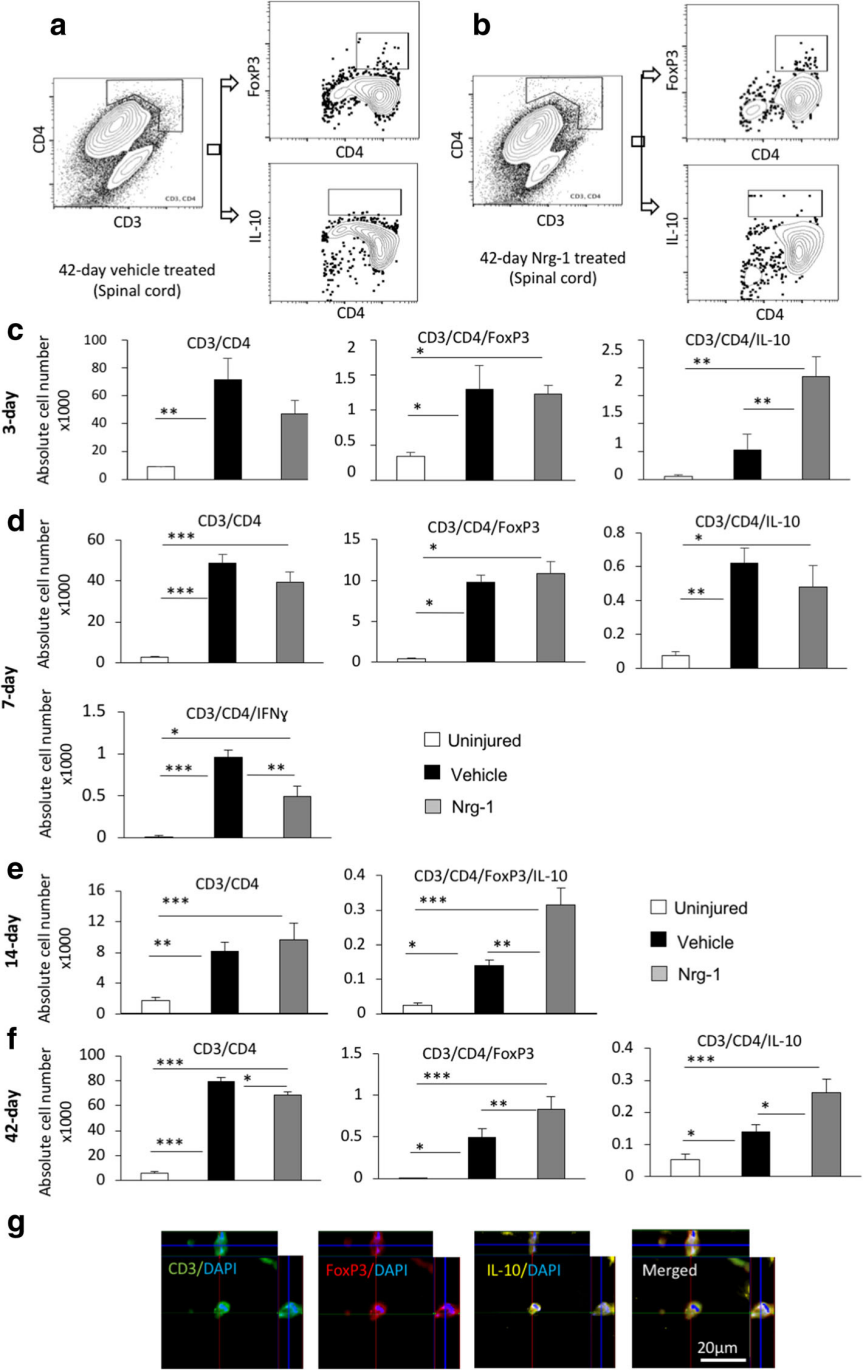
Nrg-1 promotes T_reg_ cell response in the injured spinal cord. **a**, **b** Representative images of the gating strategy for flow cytometry after singlet selection are provided for vehicle- and Nrg-1-treated groups. **c** At 3-day post-injury, the number of CD3^+^CD4^+^ T cells in the spinal cord was significantly higher in vehicle-treated animals compared to uninjured control group. There was no significant difference in the population of infiltrated helper T cells and the number of FoxP3^+^ T_reg_ cells (CD3^+^CD4^+^FoxP3^+^) between vehicle- and Nrg-1-treated groups. However, the population of IL-10 producing CD4^+^ T cells (CD3^+^CD4^+^IL-10^+^) was significantly increased in Nrg-1-treated animals in comparison to vehicle-treated group. **d** At 7 days post-SCI, despite no significant difference in the total helper and regulatory T cell populations between vehicle- and Nrg-1-treated groups, a significant reduction (1.9-fold) was observed in IFNγ producing effector T cell population in Nrg-1-treated SCI rats compared to their vehicle-treated counterparts. **e** At 14 days post-SCI, infiltrated helper T cells reached their lowest level among all examined time-points, and Nrg-1 treatment had no significant effect on the total helper T cell population. IL-10 expressing T_reg_ cells were significantly higher in both vehicle and Nrg-1 injured rats compared to uninjured animals. Nrg-1-treated animals showed a significantly higher number of T_reg_ cells in their spinal cord at 14-day time-point compared to vehicle-treated rats. **f** However, at chronic (42-day) time-point, the number of T helper cells reached a maximum, with Nrg-1-treated animals harboring a significantly decreased population of CD4^+^ T-cells in their spinal cord compared to the vehicle-treated group. Most importantly, CD3^+^CD4^+^FoxP3^+^ and CD3^+^CD4^+^IL-10^+^ regulatory T cell populations were significantly increased in Nrg-1-treated groups. **g** Immunohistochemical images show the presence of T_reg_ cells in the perilesional area (*N* = 5/group/time-point, *p < 0.05, **p < 0.01, ***p < 0.001, one-way ANOVA followed by Holm-Sidak post hoc test)

**Fig. 3:**
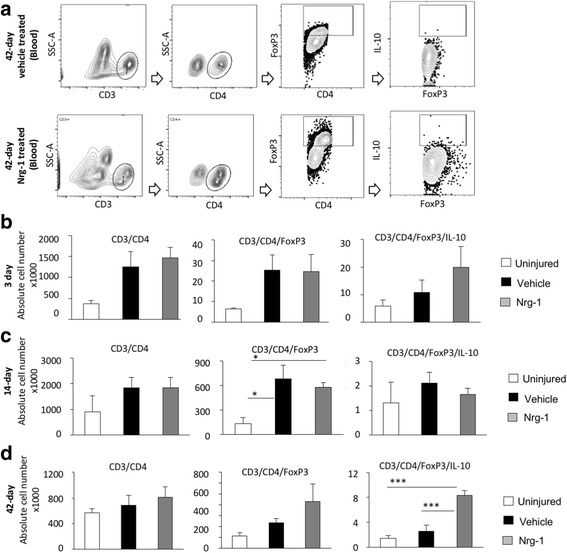
Nrg-1 treatment increases the number of circulating T_reg_ cells in the blood following chronic SCI. **a** Representative images are provided for the gating strategy for flow cytometric detection of T cells and their regulatory phenotype in the blood. **b**–**d** Flow cytometric analysis showed a slight increase in total CD3^+^CD4^+^ helper T cell population upon injury. However, at all examined time-points, the total population of helper T cells was not significantly different among any of the studied groups (*p* > 0.05). The population of FoxP3^+^ helper T cells was significantly increased at 14 days post-injury in the vehicle- and Nrg-1-treated rats as compared to the uninjured levels while there was no difference between the two injured groups. **d** At 42-day time-point, Nrg-1 treatment significantly increased the number of IL-10 expressing (CD3^+^CD4^+^FoxP3^+^IL-10^+^) T_reg_ cells compared to vehicle and uninjured groups while there was no significant difference in the total population of helper and Fox3^+^ T cells between the vehicle and uninjured groups, (*N* = 5/group/time-point, *p < 0.05, **p < 0.01, ***p < 0.001, one-way ANOVA followed by Holm-Sidak post hoc test)

**Fig. 4:**
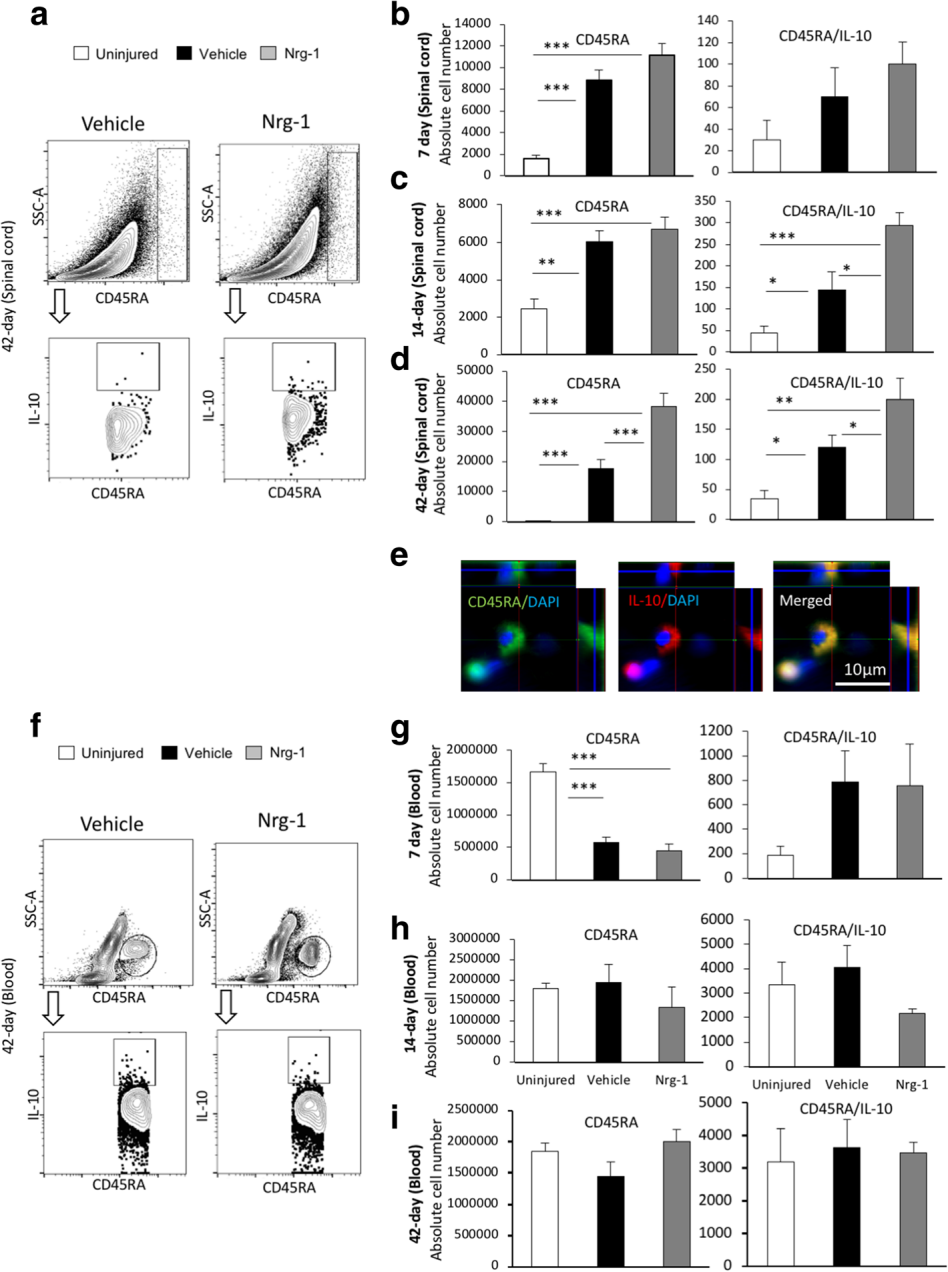
Nrg-1 treatment promotes B_reg_ cell population following SCI. **a** Representative images of the gating strategy for flow cytometry of spinal cord are provided. **b** At 7 days post-injury, the number of CD45RA^+^ B cells was significantly increased in the spinal cord without any significant difference in the total and regulatory B cell populations between vehicle- and Nrg-1-treated groups. **c** At 14-day post-SCI, a significant increase in the number of B_reg_ cells was observed in Nrg-1-treated animals compared to vehicle-treated group. **d** Chronically at 42 days post-SCI, the number of B cells in the spinal cord reached the highest level compared to all earlier time-points. Nrg-1 treatment resulted in a significant increase in the number of infiltrated B cells in the spinal cord and promoted IL-10 expressing B_reg_ cells compared to vehicle treatment. **e** Immunohistochemical analysis verified the presence of B_reg_ cells in the perilesional area of the injured spinal cord. **f** Representative images of the gating strategy for flow cytometry of blood are provided. **g** Analysis of the blood revealed a significant decline in B cell population at 7 days post-SCI without any significant change in the B_reg_ cell population at this time-point. **h**, **i** No significant change in total and regulatory B cell populations were observed in the blood at 14- and 42-day time-points. (*N* = 5/group/time-point, *p < 0.05, **p < 0.01, ***p < 0.001, one-way ANOVA followed by Holm-Sidak post hoc test)

For intracellular (cytokine) staining, following fixation, cells were incubated with permeabilizing buffer (0.1% saponin + 10% FBS in HBSS) for 30 min and incubated with a cocktail of intracellular antibodies for 30 min in dark. For FoxP3 staining, after surface antibody staining and before fixation, cells were incubated with FoxP3 fix/perm buffer for 20 min and washed in FoxP3 perm buffer (FoxP3 fix/perm buffer set, Biolegend, 421403). Cells were then incubated with perm buffer for 15 min and incubated with a cocktail of FoxP3 and IL-10 antibodies for 30 min. Finally, cells were washed with flow cytometry staining buffer (10% FBS in PBS) and reconstituted with 500 μl of this buffer and analyzed using BD FACS Canto II flow cytometer counting 200,000 events per sample. Compensation was done using single-stained beads (OneComp eBeads, 501129031, eBioscience). For each antibody panel, specific isotype controls were used to account for non-specific antibody binding (flow cytometry antibodies and their isotype controls are listed in Table 1. Gating strategies for macrophages, T cells, and B cells are shown in Figs. 1, 2, 3, and 4. Verification of antibody specificity is shown in Additional file 1: Figures S1–S3).

### Flow cytometric assessment of blood leukocytes

Upon anesthesia (described above) and before excising spinal cords, 1 ml of blood was collected through a cardiac puncture in syringes coated with and containing 0.5 ml of 100 mM EDTA. Each 200 μl of whole blood was mixed with 2 ml of RBC lysis buffer and incubated for 5 min at room temperature (420301, Biolegend). Cells were centrifuged at 600*g* for 10 min at 4 °C, and the supernatant containing lysed RBCs was discarded. This procedure was repeated one or two more times until all RBCs were lysed and a clean pellet was obtained. Cells from each animal were then pooled together, washed twice in PBS, and re-suspended in MACS buffer (PBS + 2 mM EDTA + 0.5% BSA, PH = 7.2–7.4). Leukocytes were then counted and underwent staining and flow cytometry procedures as described above.

### Tissue processing for histological studies

At each time-point, animals were deeply anesthetized (as described earlier) and were perfused transcardially with 2.5% paraformaldehyde (PFA) in 0.1 M phosphate buffered saline (PBS). Then, the spinal tissues were post-fixed in 2.5% PFA and 10% sucrose in PBS overnight. Cryoprotection continued by incubation of tissues in 20% sucrose in PBS for 2 days. A 2-cm length of the spinal cord centered at the injury epicenter was excised and embedded (Tissue-Tek® O.C.T., Electron Microscopy Sciences). Serial cross sections (35 μm thickness) were cut by a cryostat (Leica Biosystems GmbH), mounted and cover-slipped on Superfrost® Plus Micro Slides (Fischer Scientific), and stored at − 80 °C until immunostaining procedure.

### Immunohistochemical detection of macrophages and lymphocytes in the injured spinal cord

We utilized the same flow cytometry antibodies for immunohistochemical staining of T cells, B cells, and macrophages. Three tissue sections around 2 mm rostral or caudal to the epicenter were selected. Sections were washed twice with TBST (50 mM Tris, 150 mM NaCl, 0.05% Tween 20, PH 7.6) for 15 min at room temperature. Sections were blocked using 5% normal mouse serum in TBST for 1 h at room temperature and incubated with antibodies against cell surface markers for B cells (CD45RA), T cells (CD3), and macrophages (Iba-1, CD68, CD86, CD163),1:500 for Iba-1 and 1:100 for the rest of the antibodies overnight at 4 °C. Sections were then washed twice with TBST and incubated with their corresponding intracellular (IL-10 for B cells, IL-10, and FoxP3 for T cells) antibodies. FoxP3 antibody was diluted in an antibody solution containing 1% BSA, 0.1% cold water fish skin gelatin, 0.5% Triton X-100, and 0.05% sodium azide in TBS. Images were taken at × 20 and × 63 using Axio Imager M2 fluorescent microscope (Zeiss) and were processed using ZEN software (Zeiss).

### Immunohistochemical assessment of IgG and IgM in the injured spinal cord

Seven serial cross-sections spanning 7-mm length of the injured spinal cord centered at the injury epicenter were selected with 1-mm interval. Sections were blocked for 1 h with 5% mouse serum + 1% BSA + 0.3% Triton X-100 in PBS at room temperature and then incubated with a mixture of anti-IgG (Invitrogen, #31470, 1:100) and anti-IgM (eBioscience, 14-0990-82, 1:100) antibodies overnight at 4 °C. Sections were then washed three times with PBS and incubated with a mixture of Alexa Fluor® 568 donkey anti-goat (A1105, Invitrogen, 1:400) and Alexa Fluor® 647 goat anti-mouse (A21236, Invitrogen, 1:400) secondary antibodies for 1 h at room temperature. Slides were then washed with PBS three times and cover-slipped with Mowiol (Sigma) mounting medium. To avoid variability in our immunohistochemical studies, all samples were processed at the same time under the same conditions. Images were taken at × 10 magnification from whole spinal cord section using AxioImager M2 fluorescence microscope (Zeiss) under consistent exposure time as described previously [11, 17]. Fluorescence intensity was measured using ImageJ analysis software (imagej.nih.gov). After subtracting the background automatically, the immunointensity above the threshold was measured for the entire spinal cord section excluding the cavities and dura matter. To eliminate the differences in the total size of the sections, the following formula was used: normalized immunointensity of tissue section = total immunointensity of tissue section x/total area of tissue section x.

### RNA extraction and quantitative real-time PCR

At end points, the spinal cords were dissected in ice-cold artificial cerebrospinal fluid (aCSF, containing 124 mM NaCl, 3 mM KCl, 1 mM NaHPO4, 26 mM NaHCO3, 1.5 mM MgSO4, 1.5 mM CaCl2, and 10 mM glucose). Five millimeters of the spinal cord centering the injury epicenter was excised and homogenized in TRIzol reagent (Invitrogen®). RNA was extracted using an RNeasy plus mini kit (Qiagen®) and the first-strand cDNA was synthesized with 5X All-In-One RT MasterMix (ABM®). Real-time qPCR reactions were conducted with the PowerUp™ CYBR® Green Master Mix (Applied Biosystems®) in an ABI7500 fast thermocycler (Applied Biosystems) as we described before [11]. Primer information is provided in Table 2.

**Table 2:** List of primers used in this study

Rat-IFN-g-F: ATT CAT GAG CAT CGC CAA GTT C Rat-IFN-g-R: TGA CAG CTG GTG AAT CAC TCT GAT	
Rat-IL6-F: TAG TCC TTC CTA CCC CAA CTT CC Rat-IL6 R: TTG GTC CTT AGC CAC TCC TTC	
Rat-CCL11-F: TGC TGC TTG AAC ACC TTG GA Rat-CCL11-R: AGC CTG AAT ATT ACA GCT GGG T	
Rat-CCL5-F: GCA GTC GTC TTT GTC ACT CG Rat-CCL5-R: ATC CCC AGC TGG TTA GGA CT	
Rat-IL10-F: TGC GAC GCT GTC ATC GAT TT Rat-IL10-R: GTA GAT GCC GGG TGG TTC AA	
Rat-CXCL1-F: CAA TGA GCT GCG CTG TCA GT Rat-CXCL1-R: TTG AAG TGA ATC CCT GCC ACT	
Rat-CXCL2-F: AGG GTA CAG GGG TTG TTG TG Rat-CXCL2-R: CGA TCC TCT GAA CCA AGG GG	
Rat-CXCL3-F: ACA TCC AGA GCT TGA CGG TG Rat-CXCL3-R: TTG GAT GGA TCG CTG CTC TG	
Rat-CXCL10-F: CCG CAT GTT GAG ATC ATT GCC Rat-CXCL10-R: CTA GCC GCA CAC TGG GTA AA	
Rat-NFKBIZ-F: GTG GAG GCG AAG GAT CGT AA Rat-NFKBIZ-R: CAT CCA ACT GTG TCA CCC GA	

### Statistical analysis

All statistical analyses were performed using SigmaStat Software. For distance analysis in immunohistological assessments, we used two-way analysis of variance (ANOVA) followed by Holm-Sidak post hoc test. One-way ANOVA followed by Holm-Sidak post hoc was used in all flow cytometry and real-time qPCR analyses. The data was reported as a mean ± standard error of the mean (SEM), and *p* ≤ 0.05 was considered as statistically significant. Proper randomization and blinding were employed in all assessments.

## Results

### Systemic administration of neuregulin-1 alters the population of M1 and M2 macrophages following SCI

Monocyte-derived macrophages play a central role in the inflammatory response following SCI and contribute to both secondary injury mechanisms and repair processes owing to their phenotypic diversity [18]. We previously demonstrated that intrathecal infusion of Nrg-1 reduces the tissue level of pro-inflammatory cytokines in rat compressive SCI while increases IL-10 levels [13]. Here, we delivered Nrg-1 systemically and investigated the recruitment and phenotype of monocyte-derived macrophages in the injured spinal cord. Our flow cytometric assessment of uninjured spinal cord tissue showed minimal or undetectable number of macrophages (CD45^+^CD68^+^). Our SCI temporal analysis at 3, 7, 14, and 42 days post-injury, representing acute, subacute, and chronic stages of injury, showed infiltration of CD45^+^CD68^+^ macrophages to the lesion as expected. We found a significant 13.6- and 184-fold increase in the CD45^+^CD68^+^ cell population at 3 and 7 days post-injury, respectively (Fig. 1c). While remained significantly higher than uninjured control animals, the number of macrophages was decreased to a lower level at 14 days post-injury and onwards up to 42 days, the latest examined time-point (Fig. 1c–f). Nrg-1 treatment significantly increased the population of infiltrated macrophages at 3 days post-injury (1.9-folds) with no significant effect on their population at 7 and 14-day time-points. However, chronically at 42 days post-SCI, Nrg-1 treatment led to a significantly higher number of CD45^+^CD68^+^ macrophages in the spinal cord compared to vehicle treatment (1.34 times).

We next performed immunophenotyping to determine whether Nrg-1 alters subpopulations of macrophages in the injured spinal cord. Our analysis of pro-inflammatory (M1) macrophages (CD45^+^CD68^+^CD86^+^) comparing uninjured and vehicle-treated injured rats showed an overall increase in M1 population at all examined time-points following SCI. This increase was statistically significant at 7, 14, and 42 days post-SCI (*p* < 0.01) (Fig. 1c–f). Compared to the baseline of SCI in vehicle-treated groups, Nrg-1 treatment had no apparent effect on the population of M1 macrophages at 3, 7, and 14-day time-points. However, the population of M1 macrophages was significantly increased (1.6-fold) in Nrg-1-treated animals at the chronic 42-day time-point compared to the vehicle group (*p* < 0.01) (Fig. 1f). Our phenotypic analysis revealed that Nrg-1 significantly increased the number of immunomodulatory (CD45^+^CD68^+^CD163^+^) M2 macrophages (3-fold) as well as their IL-10 expressing subpopulation (CD45^+^CD68^+^CD163^+^IL-10^+^, 4.1-fold) at 3-day time-point. Moreover, we observed an increasing trend in the number of M2 macrophages at 7, 14, and 42-day time-points following Nrg-1 treatment, although these changes were not statistically significant. Using immunohistochemistry, we verified and confirmed the presence of M1 (CD68^+^CD86^+^) and M2 (CD68^+^CD163^+^) macrophages in the perilesional area in the injured spinal cord (Fig. 1g). Altogether, our results indicate that systemic Nrg-1 treatment following SCI augments the recruitment of blood-borne macrophages to the spinal cord tissue at acute and chronic stages with an increase in M2 phenotype at acute (3-day) and M1 phenotype during chronic (42-day) stage of SCI (Fig. 1c, f, verification of antibody specificity and gating strategy are shown in Additional file 1: Figure S1).

### Neuregulin-1 promotes a regulatory T cell response in the blood and spinal cord following SCI

We next studied how systemic Nrg-1 infusion affects T cell response in the peripheral blood and within the injured spinal cord. T cells orchestrate the adaptive immune response following SCI [19] with differential roles through their diverse production of pro- and anti-inflammatory cytokines and chemokines [20]. Using the same time-points described above, our flow cytometric analysis of the spinal cord tissue in baseline vehicle SCI rats showed an overall significant increase in the population of infiltrated helper T cells (CD3^+^CD4^+^) in the injured spinal cord at 3, 7, 14, and 42 days post-injury by 7.7-, 16.3-, 4.5-, and 13.5-folds, respectively (*p* < 0.01 at 3-day and *p* < 0.001 at all later time-points). Interestingly, while CD3^+^CD4^+^ helper T cells were significantly present at all stages of SCI, we found a decline in their number subacutely on day 14 post-injury. However, this decline was followed by an increase in their population reaching a maximum at the chronic stage of SCI on day 42 (13.5 times increase) (*p* < 0.001) (Fig. 2c–f). The biphasic baseline pattern of T cell recruitment after SCI is in agreement with previous studies [1]. Nrg-1 treatment had no apparent effect on the population of helper T cells in acute and subacute SCI, whereas it significantly reduced their recruitment in the chronic SCI at the 42-day time-point.

We next performed immunophenotypic analysis within the CD3^+^CD4^+^ helper T cell population to determine whether Nrg-1 treatment can alter T cell subsets in the injured spinal cord. Our analysis showed that SCI promotes T regulatory (T_reg_) (CD3^+^CD4^+^FoxP3^+^ and CD3^+^CD4^+^IL-10^+^) subpopulation after SCI. Nrg-1 treated SCI animals had a significantly higher population of T_reg_ cells at 3 days (3.5-folds, *p* < 0.01), 14 days (5.6-folds, *p* < 0.01), and 42 days (1.8-folds, *p* < 0.05) time-points compared to vehicle-treated (Fig. 2c–f). Analysis of T effector (T_eff_) cell subpopulation (CD3^+^CD4^+^IFN-γ^+^) also showed a remarkable positive effect for Nrg-1 in modulating T cell response in subacute SCI. At 7 days post-injury, compared to the uninjured group, SCI induced a significant increase in T_eff_ cells within the injured spinal cord of vehicle-treated rats (100-fold, *p* < 0.001) which was significantly reduced by Nrg-1 treatment to nearly 50% (Fig. 2d). At other time-points, no significant change in T_eff_ cell subpopulation was detected under Nrg-1 treatment (data not shown). Our immunohistochemical assessments verified the presence of CD3^+^FoxP3^+^IL10^+^ T_reg_ cells in the perilesional area of the injured spinal cord (Fig. 2g).

We further investigated the systemic effects of Nrg-1 on T cell recruitment and phenotype in the blood following SCI by flow cytometric analysis (Fig. 3). We observed an increasing trend in the population of helper T cells (CD3^+^CD4^+^) in the blood of SCI animals. However, this increase was not statistically significant at any of the examined time-points, and Nrg-1 treatment had no apparent effect on the population of helper T cells after SCI (Fig. 3b–d). Our analysis of CD3^+^CD4^+^FoxP3^+^IL10^+^ cells also showed no change in the population of Treg cells in the blood under the baseline of SCI. While Nrg-1 treatment had no noticeable effect on T_reg_ cell population in the blood at 3 and 14 days post-SCI, it stimulated a significant (3.7-fold) increase in the number of IL-10 expressing T_reg_ cells (CD3^+^CD4^+^FoxP3^+^IL-10^+^) chronically at 42 days post-injury compared to their vehicle-treated counterparts (Fig. 3d, verification of antibody specificity and gating strategy are shown in Additional file 1: Figure S2). In conclusions, our analysis of T lymphocyte response demonstrates the ability of Nrg-1 to promote the population of immune-modulatory T_reg_ cells in the blood and spinal cord after SCI.

### Nrg-1 treatment is associated with an increase in regulatory B cell phenotype following SCI

B cells contribute to SCI secondary tissue damage after SCI by producing cytokines and autoantibodies [10, 21]. Despite their detrimental roles in tissue degeneration, B cells have the ability to positively modulate immune response through adopting an IL-10 producing immune regulatory (B_reg_) phenotype [22]. Our flow cytometric analysis showed that SCI triggers the recruitment of B cells to the spinal cord. We found a significant increase in the total number of CD45RA^+^ B cells in the vehicle-treated injured spinal cord at all examined time-points compared to uninjured group (5.4-fold at 7-day, 2.4-fold at 14-day, and 61-fold at 42-day) (*p* < 0.01) (Fig. 4b–d). While Nrg-1 treatment did not alter B cell infiltration at the subacute 7-day and 14-day time-points, it significantly induced B cell recruitment to the injured spinal cord chronically at the 42-day time-point (2.1-fold, *p* < 0.001) compared to vehicle-treated group (Fig. 4d). Analysis of B_reg_ cells subpopulation (CD45RA^+^IL-10^+^) in the spinal cord tissue showed the ability of Nrg-1 treatment to significantly promote IL-10 producing B_reg_ population at 14 days (2-folds) and 42 days (1.6-folds) post-SCI (*p* < 0.05) compared to vehicle treatment (Fig. 4c, d). Presence of CD45RA^+^IL10^+^ cells in the perilesional area of the injured spinal cord was verified in our immunohistochemical analysis (Fig. 4e). Interestingly, our flow cytometric analysis showed a significant threefold reduction in CD45RA^+^ B cell population in the blood at 7-day post-SCI in both vehicle- and Nrg-1-treated SCI rats compared to uninjured control group (*p* < 0.001), which was recovered to the normal baseline by the 14-day time-point (Fig. 4g, h). This observation is in line with other studies that reported a transient cessation of B lymphopoiesis in the bone marrow following SCI [23]. Nonetheless, there was no significant difference in the number of circulating B_reg_ cells in the blood between vehicle- and Nrg-1-treated animals at any examined time-point (Fig. 4g–i, verification of antibody specificity and gating strategy are shown in Additional file 1: Figure S3). Altogether, our data show that systemic administration of Nrg-1 induces a B_reg_ cell response following SCI only at the level of spinal cord tissue without affecting the phenotype of circulating B cells.

### Nrg-1 treatment positively modulates inflammatory cytokine expression following SCI

**Fig. 5:**
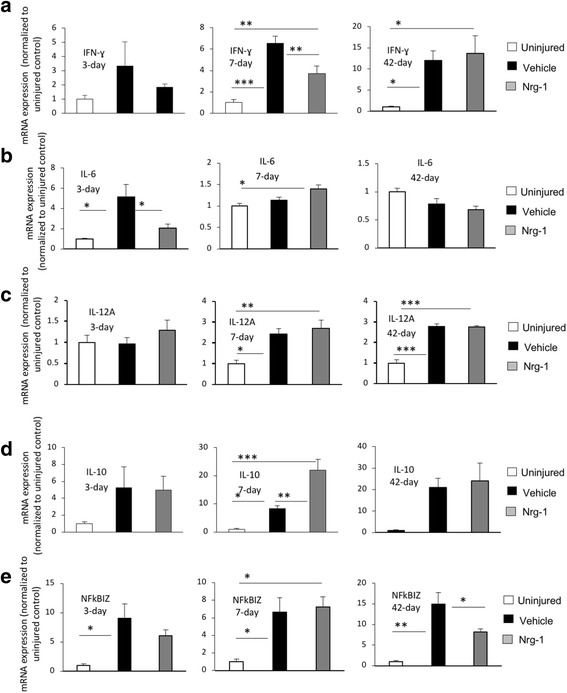
Nrg-1 treatment regulates inflammatory cytokines in the injured spinal cord. **a** Transcript analysis of the spinal cord tissue using real-time qPCR released a significant increase in expression of IFN-γ at 7-day post-injury (*p* < 0.001). Nrg-1 treatment significantly decreased the SCI-induced expression of IFN-γ in the injured spinal cord tissue. **b** Expression of IL-6 was significantly increased at 3 days post-injury, which was significantly attenuated by Nrg-1 treatment (*p* < 0.05). No significant difference in IL-6 expression was observed between vehicle- and Nrg-1-treated groups at 7- and 42-day time-points. **c** IL-12A was significantly increased at 7 days post-injury and remained significantly elevated until 42 days. Nrg-1 had no significant effect on IL-12A expression at any examined time-point. **d** Transcript levels of IL-10 were significantly increased at 7 days post-injury. Interestingly, Nrg-1-treated animals showed a significantly higher expression of IL-10 compared to the vehicle group at this time-point. No significant change in IL-10 expression was observed at any other examined time-points. **e** NFkBIZ transcript level was significantly elevated at 3 days and reached its maximum at 42 days post-injury. Nrg-1 treatment significantly attenuated NFkBIZ expression chronically at 42 days post-SCI (*N* = 4/group/time-point, *p < 0.05, **p < 0.01, ***p < 0.001, one-way ANOVA followed by Holm-Sidak post hoc test)

We previously showed that intrathecal infusion of Nrg-1 reduces the level TNF-α and IL-1β in the injured spinal cord [13]. Here, we extended these initial findings to investigate the effect of systemic Nrg-1 treatment on the expression of several key inflammatory cytokines involved in the regulation of immune response following SCI including IFN-γ, IL-6, IL-12, and IL-10 [24–27]. Our real-time qPCR analysis of IFN-γ expression, a pro-inflammatory cytokine involved in M1 macrophage activation, and cytotoxic T cell proliferation [28, 29], showed a significant 6.5- and 12-fold increase at 7-day and 42-day time-points, respectively, in vehicle-treated rats as compared to uninjured group (Fig. 5a). Interestingly, Nrg-1 treatment significantly attenuated IFN-γ expression subacutely at 7 days post-SCI by 47% compared to vehicle treatment with no apparent effect at the 42-day chronic time-point (*p* < 0.01) (Fig. 5a). Next, we assessed the expression of the pro-inflammatory cytokine IL-6 which is known to induce cellular injury and tissue degeneration following SCI [30]. IL-6 expression was transiently and significantly increased (5.1-fold) at 3 days after injury in vehicle-treated animals compared to uninjured control group and returned to its normal baseline levels at 7 days post-SCI and onwards (Fig. 5b). Nrg-1-treated animals showed a significant 2.4-fold reduction in IL-6 expression in their injured spinal cord at acute 3-day time-point (*p* < 0.05) (Fig. 5b). There was no significant difference in IL-6 expression between the vehicle- and Nrg-1-treated animals at any later time-point (*p* > 0.05) (Fig. 5b). Expression of IL-12A, a pro-inflammatory cytokine involved in microglia/macrophage activation with both beneficial and detrimental roles [26, 31], was also significantly elevated at subacute 7-day post-injury (2.4-fold) which remained upregulated up to the chronic 42-day time-point (2.7-fold) (*p* < 0.05) (Fig. 5c). Nrg-1 treatment was not associated with any significant change in IL-12A expression at any stage after SCI (Fig. 5c).

Given Nrg-1 promoted an IL-10 regulatory phenotype in macrophages and lymphocytes, we also assessed IL-10 transcript expression. Seven days after injury, the expression of IL-10 increased significantly (8.3-fold) in the injured spinal cord (Fig. 5d). In agreement with our flow cytometry data, Nrg-1 treatment resulted in a significantly higher (2.6-fold) expression of IL-10 compared to vehicle treatment (Fig. 5e). Change in IL-10 expression was more pronounced in subacute stage of SCI (Fig. 5d).

Finally, we investigated the expression of NFκB inhibitor zeta (NFkBIZ or IκBζ), an atypical IκB which is known to be an essential activator of IL-6 expression and acts as a transcription co-activator of pro-inflammatory cytokines such as IL-12 [32, 33]. The expression of NFκBIZ was significantly increased at 3 and 7 days post-injury (9- and 6.6-fold, respectively) and reached its maximum at 42-day time-point (14.9-fold) (Fig. 5e). This elevation was reduced significantly (45%) by Nrg-1 treatment chronically at 42 days post-injury (Fig. 5e). NFκBIZ expression was also reduced by Nrg-1 treatment acutely; however, the effect was not statistically significant. Altogether, these results show a positive role for Nrg-1 in regulating inflammatory cytokine profile of the injured spinal cord tissue which seemed to be modulated, at least in part, by NFκB pathway following SCI.

### Pro-regenerative modulation of chemokine expression by Nrg-1 treatment following SCI

In addition to cytokine assessment, we sought to determine the effect of systemic Nrg-1 treatment on chemokine profile in the spinal cord tissue. We focused on C-C motif ligands (CCL) 5 and 11 and C-X-C motif ligands (CXCL) 1, 2, 3, and 10 that are involved in the pathophysiology of SCI [34–39].

#### Effects of Nrg-1 on the expression of CCL 5 and 11

**Fig. 6:**
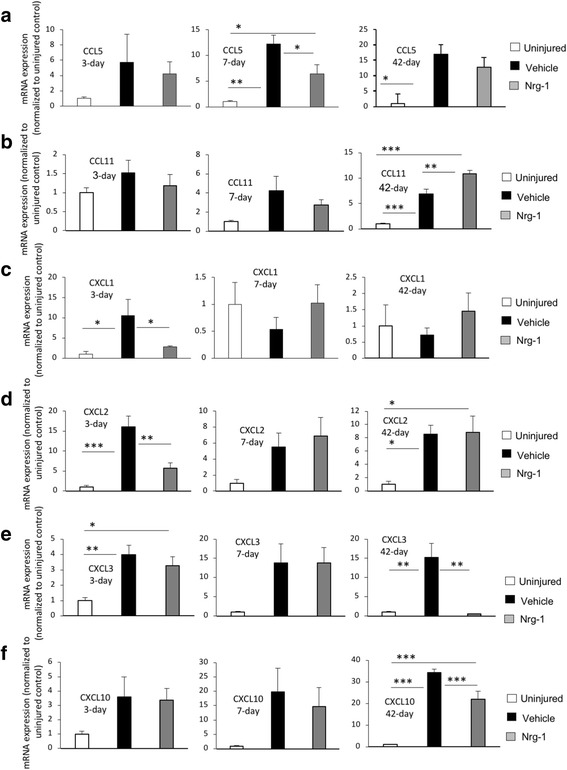
Nrg-1 treatment positively modulates chemokine expression following SCI. **a** Analysis of chemokine expression in the spinal cord tissue was performed using real-time qPCR. There was a significant increase in the expression of pro-inflammatory chemokine CCL5, at 7-day post-injury, which was significantly reduced by Nrg-1 treatment. CCL5 mRNA level reached its maximum level at 42 days post-injury. However, no significant difference was found between vehicle- and Nrg-1-treated animals at this time-point. **b** Although the expression of CCL11, an immune modulatory chemokine, was not significantly changed at 3 and 7 days post-injury, it underwent a significant increase chronically at 42-day time-point. Nrg-1 treatment was able to significantly increase CCL11 expression compared to vehicle-treated group. **c**, **d** Expression of pro-inflammatory chemokines CXCL1 and CXCL2 was significantly elevated at 3-day post-injury. Nrg-1 treatment significantly reduced CXCL1 and CXCL2 expressions at this time-point. The overall tissue level of CXCL1 mRNA reached baseline levels at 7-day post-injury and remained stable until 42 days post-injury. CXCL2 expression, however, remained elevated at 7 and 42 days post-injury compared to uninjured group (significant 8.5-fold increase at 42-day). Nrg-1 treatment had no significant effect on CXCL2 expression at 7 and 42-day time-points. **e** Expression of the pro-inflammatory chemokine CXCL3was significantly upregulated 3 days after injury and reached its maximum levels chronically at 42 days post-SCI (*p* < 0.01). Nrg-1-treated animals showed a significant reduction in CXCL3 expression at 42-day time-point (31 times). **f** CXCL10 expression was not significantly changed following an injury at 3 and 7 days post-SCI, while it was significantly increased in the vehicle-treated group compared to un-injured animals at 42 days. Nrg-1 significantly decreased CXCL10 expression at this time-point (*p* < 0.001) (*N* = 4/group/time-point, *p < 0.05, **p < 0.01, ***p < 0.001, one-way ANOVA followed by Holm-Sidak post hoc test)

C-C motif chemokine 5 (CCL5 or RANTES) is produced by resident glial and peripherally recruited immune cells including T cells, macrophages, and astrocytes and is known to induce neuroinflammation by attracting leukocytes to the site of injury [40, 41]. The increased CCL5 level has been associated with microvascular dysfunction and tissue damage following CNS injury [42]. At 3-day post-SCI, the expression of CCL5 was increased (Fig. 6a). However, this increase was not statistically significant, and Nrg-1 treatment had no apparent effect on CCL5 expression acutely. At 7 and 42 days post-SCI, CCL5 expression in the spinal cord was remarkably increased by 12- and 16-folds, respectively, compared to uninjured animals (*p* < 0.01). Nrg-1-treated animals demonstrated a significant 47% reduction in CCL5 expression at 7 days post-SCI with an insignificant 24% decline at 42-day chronic time-point (*p* < 0.01) (Fig. 6a).

CCL11 or eotaxin-1 is a small chemokine that is mainly produced by macrophages and neurons with positive effects in CNS injury including tightening the blood-brain barrier and fostering a Th2 immunomodulatory response [39]. CCL11 is also considered a pro-regenerative chemokine by promoting proliferation and migration of neural progenitor cells [43]. Our qPCR analysis of the spinal cord tissue at 3- and 7-day time-points in both vehicle- and Nrg-1-treated animals showed no significant change in CCL11 expression (Fig. 6b). However, at the chronic 42-day time-point, SCI baseline expression of CCL11 reached a significant 6.8-fold increase compared to the uninjured control group (Fig. 6b). Interestingly, Nrg-1 treatment induced a significant 37% increase in CCL11 expression compared to vehicle treatment (Fig. 6b).

#### Effects of systemic Nrg-1 treatment on the expression of CXC family of chemokines

CXCL1, 2, 3, and10 play a substantial role in SCI neuroinflammation and induction of neuropathic pain [36, 44]. CXCL1 and CXCL2 are produced by mast cells and macrophages and control neutrophil recruitment during tissue inflammation [45]. Using qPCR, we observed a significant increase in the expression of CXCL1 and CXCL2 at 3 days post-injury (10- and 16-folds, respectively). Nrg-1 treatment significantly reduced the expression of both chemokines acutely (74 and 64% decrease, respectively) (Fig.6c, d). There was no significant change in the expression of CXCL1 at subacute and chronic time-points. However, the expression level of CXCL2 was significantly increased at the 42-day chronic time-point compared to uninjured control group (8.5-fold), although Nrg-1-treated animals showed no significant change in the expression of CXCL2 compared to vehicle-treated group at this time-point (Fig. 6d). Next, we assessed the expression of CXCL3, which is a ligand for CXC chemokine receptor (CXCR) 2 [34]. It has been shown that inhibition of CXCR2 attenuates neuroinflammation and improves tissue preservation following SCI [34]. CXCL3 expression was increased significantly at the acute 3-day (3.9-fold) and chronic 42-day (15.2-fold) post-injury (Fig. 6e). Nrg-1 treatment remarkably decreased (96%) the expression of CXCL3 at the 42-day time-point.

CXCL10 is another member of CXC family of chemokines which is known for its distinct role in T cell recruitment and exacerbation of secondary tissue degeneration following SCI [35]. Neutralization of CXCL10 has been associated with better structural and functional outcomes following SCI [35]. Following injury, expression of CXCL10 was increased as early as 3 days post-SCI. However, this increase was only statistically significant at the chronic 42-day post-injury (34-fold increase) compared to uninjured group (Fig. 6f). Similar to the other examined CXCL chemokines, Nrg-1-treated animals showed a significant 36% decrease in CXCL10 expression chronically (Fig. 6f). Taken together, these results demonstrate that Nrg-1 treatment can positively modulate the repertoire of cytokines and chemokines in the spinal cord tissue following SCI. Importantly, our expression data, in agreement with our flow cytometry data, indicate a more prominent role for Nrg-1 in modulating neuroinflammation at the chronic stage of SCI.

### Systemic Nrg-1 treatment decreases IgM and IgG deposition in the injured spinal cord acutely

**Fig. 7:**
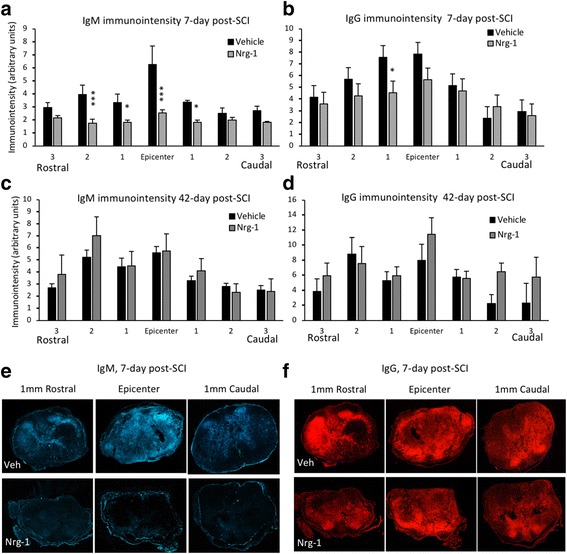
Nrg-1 therapy significantly attenuates antibody deposition in the injured spinal cord tissue at subacute stage of SCI. **a**–**d** Immunohistochemical assessment of injured spinal cord tissue for IgM and IgG deposition was performed at the epicenter and perilesional areas at subacute (7 days) and chronic (42 days) stages of SCI. **a**, **c** Analysis of IgM immunointensity showed a significantly lower IgM deposition in Nrg-1-treated animals compared to vehicle-treated group at 7-day post-SCI. This decrease was significant at the epicenter, 1 mm caudal and 1 and 2 mm rostral to the injury site. No significant difference in IgM deposition was detected at 42-day time-point. **b**, **d** Comparison of IgG immunointensity between the vehicle- and Nrg-1-treated animals revealed a significant decrease in IgG deposition only at 1 mm rostral to the epicenter at 7-day post-injury. No significant difference was detected in IgG deposition between the vehicle- and Nrg-1-treated groups at 42-day time-point. **e**, **f** Representative images are provided at the epicenter and 1 mm rostral and caudal to the lesion center (*N* = 4/group/time-point, *p < 0.05, **p < 0.01, ***p < 0.001, two-way ANOVA followed by Holm-Sidak post hoc test)

Following SCI, autoantibodies are produced by B cells against spinal cord neoepitopes and contribute to neuroinflammation and secondary tissue damage [21]. Since we identified a role for Nrg-1 in regulating B cell response in SCI, we sought to study antibody deposition in the injured spinal cord. We performed immunohistochemical analysis of IgM and IgG in the injured spinal cord, spanning 6 mm of injured tissue around the injury epicenter rostrally and caudally. At 7 days post-injury, we found a significant reduction in IgM deposition in the Nrg-1-treated group as compared to vehicle-treated rats. This reduction was significant at the epicenter (163-fold), 1- and 2-mm rostral (1.8- and 2.2-fold, respectively) as well as 1-mm caudal (1.8-fold) points to the injury epicenter (*p* < 0.05, two-way ANOVA, *N* = 5/group) (Fig. 7a). In a similar immunointensity analysis, we also found a reduction in IgG deposition in the spinal cord of Nrg-1-treated animals compared to vehicle-treated group. This reduction was statistically significant at 1 mm rostral to the injury epicenter (Fig. 7b). Analysis of IgG and IgM deposition at the chronic 42-day time-point showed no significant difference between vehicle- and Nrg-1-treated groups (Fig. 7c, d). These results show the ability of Nrg-1 to reduce antibody deposition at subacute stage of SCI and a plausible mechanism through which Nrg-1 improved neural tissue preservation in our previous studies.

## Discussion

In the current study, using a clinically relevant model of compressive/contusive SCI in rats, we have identified a novel positive immunomodulatory role for Nrg-1 in SCI. Systemic Nrg-1 therapy augments regulatory populations of macrophages, T cells, and B cells both peripherally and in the injured spinal cord tissue during the acute and chronic phases of SCI. Moreover, Nrg-1 treatment promotes pro-regenerative immune mediators such as IL-10 and CCL11 while attenuating pro-inflammatory cytokines and chemokines, IL-6, IFN-γ, CXCL1, CXCL2, and CXCL3. Importantly, Nrg-1 positively regulates B cell activity and moderates SCI-induced deposition of IgG and IgM in the spinal cord. To our knowledge, this is the first study that investigates the impact of Nrg-1 on neuroinflammation in traumatic SCI. Importantly, we have demonstrated the potential of systemic Nrg-1 as a new promising immunomodulatory strategy for traumatic SCI in a clinically relevant model. Of note, the Nrg-1 treatment used in our study is shown to pass the blood-brain and spinal cord barrier and enter the central nervous system (CNS) readily [46] and importantly has shown therapeutic efficacy in previous studies by our group and others [13, 47].

Nrg-1 and ErbB network is known for its critical role in the developing central and peripheral nervous systems [48]. In the spinal cord, while the implication of Nrg-1 in neural differentiation and myelination is established during development [49, 50], our knowledge on the role of Nrg-1 in SCI pathophysiology and neuroinflammation is still in its infancy. In recent years, work by our group and others has begun unraveling the importance of Nrg-1 in the pathologic CNS [11, 13–15]. We originally identified that Nrg-1 protein expression is severely depleted in acute traumatic SCI and remains down-regulated chronically [11]. We established a close correlation between Nrg-1 dysregulation and impaired endogenous precursor response after SCI [11]. Restoration of Nrg-1 was sufficient to activate an endogenous repair program that promoted oligodendrocyte replacement and white matter repair following SCI [11, 13]. A recent study has also shown an essential role for Nrg-1 in Schwann cell driven remyelination after SCI that is known to be instrumental for endogenous myelin repair in the injured spinal cord [12]. Interestingly, we found that Nrg-1 treatment exerts a prominent neuroprotective effect on oligodendrocytes and axons after SCI [11], which complements the recent reports showing increased neuronal survival following ischemic brain insult [15]. In addition to the anticipated impact of Nrg-1 on oligodendrocytes and myelination, our recent in vitro and in vivo studies uncovered a novel role for Nrg-1 in regulating astrocyte response to injury and evolution of the glial scar in the injured spinal cord [13]. More specifically, Nrg-1 treatment had a remarkable effect on matrix remodeling in the glial scar by moderating the SCI-induced upregulation of chondroitin sulfate proteoglycans (CSPGs), a key regulator of SCI pathophysiology and a master inhibitor of repair processes [51]. We unraveled that Nrg-1 mediates its effects through activation of ErbB2/3 complex in glial cells and an increase in phosphorylation of Erk1/2 and STAT3 pathways following SCI [13]. Most importantly, we showed that Nrg-1 treatment improves neurobehavioral recovery in a dose-dependent manner after SCI [13]. Interestingly, these studies also provided the initial evidence for immunomodulatory effects of Nrg-1, which seems to be an underpinning mechanism for its beneficial effects in SCI.

We and others have demonstrated that Nrg-1 positively influences microglia in vitro and attenuates their response to stressful conditions as evidenced by the reduced production of pro-inflammatory mediators such as nitric oxide (NO), IL-1β, and TNF-⍺ [13, 52]. In rat SCI, we found that intrathecal Nrg-1 treatment can remarkably enhance an IL-10 dominant cytokine balance [13] that is shown to be beneficial for SCI repair processes such as remyelination [53]. Several immune regulatory populations such as T_reg_ cells, B_reg_ cells, and M2 microglia/macrophages produce IL-10 after SCI suggestive of a broader role for Nrg-1 in modulating neuroinflammation [54–56]. Notably, T and B lymphocytes, macrophages, and microglia express Nrg-1 receptors [13, 57–59], and therefore, all these populations can potentially be affected by the dysregulation of Nrg-1 signaling following SCI. To date, the role of Nrg-1 in modulating the innate and adaptive immune response in SCI has remained elusive. In this study, we employed systemic Nrg-1 delivery to understand how Nrg-1 influences the recruitment and function of immune cells not only within the injured spinal cord tissue but also in the peripheral blood. Additionally, systemic delivery provides a more clinically relevant therapeutic strategy for SCI.

Our immunophenotypic investigation of the injured spinal cord tissue revealed that systemic Nrg-1 increases macrophage infiltration into the injured spinal cord, which seems to be temporally regulated. We found that Nrg-1 promotes the recruitment of macrophages acutely and chronically with no apparent effect at the subacute stage. Interestingly, previous studies on acute peripheral nerve injury have also shown that intrathecal infusion of Nrg-1 induces recruitment and proliferation of resident microglia in the dorsal horn where injured sensory afferents enter the spinal cord [60, 61]. Increased microgliosis and macrophage proliferation in response to over-activation of Nrg-1 and ErbB receptors have been also shown in direct in vitro studies [60, 61]. Although Nrg-1-induced microgliosis has been associated with neuropathic pain in peripheral nerve injury [60, 61], our previous studies in rat SCI showed no significant change in pain sensation following Nrg-1 treatment [13]. It is noteworthy that Nrg-1 treatment in our SCI studies augments the downregulated levels of Nrg-1 in the injured spinal cord as opposed to over-activating its signaling. Interestingly, although Nrg-1 increased the population of macrophages in acute SCI, this increase mainly reflected an elevation in the subpopulation of alternatively activated M2 macrophages (CD45^+^CD68^+^CD163^+^IL10^+^) with no change in the population of classically activated pro-inflammatory M1 (CD45^+^CD68^+^CD86^+^) macrophages. Notably, the increase in M2 macrophage population at the acute stage of SCI is in agreement with our previous cytokine study in SCI where we observed elevated levels of M2 markers such as arginase-1 and IL-10 under Nrg-1 therapy [13]. M2 macrophages are shown to promote oligodendrocyte differentiation, survival, and remyelination [62] and are associated with overall tissue preservation and improved recovery of function following SCI [63]. In the chronically injured spinal cord tissue, however, it was intriguing that Nrg-1 treatment more prominently promoted the population of M1 macrophages with a concurrent increase in IL-10 expressing M2 subpopulation. Further studies are required to fully elucidate the underlying mechanisms of Nrg-1 in macrophage regulation.

We show that Nrg-1 treatment provided a more balanced chemokine profile after SCI. Nrg-1 elevated the expression level of CCL11 in chronic SCI. CCL11 is produced by microphages/microglia, astrocytes, and pericytes [64–66]. Similar to IL-10, CCL11 is known to have anti-inflammatory function and protects neural tissue during inflammatory process [39]. CCL11 also increases migration and proliferation of neural precursor cells following cerebral ischemic injury [43], which is a prerequisite for endogenous cell replacement. We additionally showed that Nrg-1 attenuated the expression of CXCL1 in acute SCI. This chemokine is produced by mast cells, macrophages, and astrocytes and contributes to neutrophil recruitment and exacerbate neuroinflammation following CNS injury [45]. Interestingly, CXCL1 is known to be regulated by IL-6 [67], an inflammatory cytokine produced by macrophages, microglia, and astrocytes in SCI [68, 69]. Thus, these data suggest a correlation between downregulation of CXCL1 and reduction in IL-6 expression by Nrg-1 in our acute SCI studies. Notably, inhibition of IL-6 signaling is associated with reduced glial scarring and improves functional recovery [70] and neuropathic pain following SCI [25]. Our chemokine profiling also showed the potential of Nrg-1 in attenuating the expression of CXCL2 and CXCL10. CXCL2 is known to induce neuronal injury in motoneuron cultures [71] supportive of a neuroprotective role for Nrg-1 in CNS injury. CXCL10, also known as interferon-gamma-induced protein 10, is a pro-inflammatory chemokine produced by macrophages, fibroblasts, astrocytes, and endothelial cells upon IFN-ɣ stimulation [72]. Neutralization of CXCL10 has been associated with decreased secondary tissue degeneration and improvement of functional recovery in murine SCI [35]. Taken together, our chemokine analysis indicates that while Nrg-1 promotes the overall recruitment of macrophages into the injured spinal cord, it activates a phenotype in macrophages that can facilitate repair processes following SCI.

A novel finding in our study is the modulatory effect of Nrg-1 on T cells after SCI. We have provided the first evidence that Nrg-1 treatment remarkably enhances a T_reg_ response in acute, subacute, and chronic SCI. Most interestingly, Nrg-1 exerts its beneficial effects systemically by increasing the population of circulating T_reg_ cells in the bloodstream in chronic SCI. T_reg_ cells play pivotal roles in regulating an adaptive immune response and preventing autoimmune reactions [73]. Ablation of T_reg_ cells elicits an intensive T_eff_ response that induces neuronal death following CNS injury [54]. In our study, we showed the ability of systemic Nrg-1 treatment to suppress the population of pro-inflammatory CD3^+^CD4^+^IFN-γ^+^ effector T cells and reduce IFN-γ^+^ within the injured spinal cord tissue at subacute SCI. Of note, IFN-γ is primarily produced by lymphocytes including effector Th1 cells and promotes classic macrophage activation [29] and neuropathic pain [24]. Importantly, IFN-γ is implicated in inducing white matter degeneration and necrosis following cerebral ischemia/reperfusion injury and increasing the susceptibility of neurons to apoptosis [74, 75]. SCI studies have shown that IFN-γ directs its degenerative effects by promoting proliferation of cytotoxic T cells [8, 76]. Indeed, reduction in IFN-γ expression in subacute SCI under Nrg-1 treatment in this study provides an underlying mechanism by which Nrg-1 ameliorated white matter degeneration in the injured spinal cord in our previous studies [11, 13]. Nrg-1 also induced a reduction in the expression of CCL5 (RANTES), a pro-inflammatory chemokine produced by astrocytes as well as T cells upon macrophage stimulation [68, 77]. This observation correlates well with the increased M2 macrophages and decreased population of effector T cells that we observed in our immunophenotypic studies. Increase in CCL5 is implicated in microvascular dysfunction following CNS injury [42]. Altogether, our observations indicate that Nrg-1 is a positive modulator of T cell response after SCI. Therefore, increasing the deficient levels of Nrg-1 after injury has therapeutic value to augment a T regulatory immune response during the repair processes following SCI.

SCI also elicits a B cell response in the spleen and bone marrow characterized by increased B cell number and elevated serum immunoglobulin levels [4]. Our findings provide new evidence that Nrg-1 promotes recruitment and regulatory phenotype of B cells in the injured spinal cord with a more pronounced effect at the chronic stage of injury. Similar to T cells, the increase in B cell response mainly represented a rise in the B_reg_ population in response to Nrg-1 treatment. Interestingly, in contrast to the spinal cord, the number of B cells was initially dropped in the blood after SCI which was independent of Nrg-1 treatment. This observation is consistent with previous reports that showed SCI induces an initial cessation of B cell production in the bone marrow [23]. There is also evidence that B cells undergo extensive apoptosis following SCI. B cells are the integral component of the adaptive humoral immune response and capable of producing a wide variety of cytokines [10]. Following SCI, B cells produce autoantibodies against spinal cord tissue that can aggravate the secondary injury processes through complement and Fc receptor (FcR)-dependent mechanisms [21]. Similar to other immune cells, phenotype and function of B cells can be regulated by the signaling molecules and cytokines available in their microenvironment [10]. B_reg_ cells support repair processes as they can suppress activation of helper T cell and their production of TNF-α and IFN-ɣ as well as TNF-α production by monocytes [78–80]. Importantly, B_reg_ cells play essential roles in the formation and maintenance of T_reg_ cell population [81]. Moreover, B_reg_ cell-mediated IL-10 production has been shown to limit tissue damage and improve recovery of function in a murine model of brain ischemia [22]. For the first time, we uncovered that Nrg-1 attenuates antibody deposition in the injured spinal cord. Our analysis of IgG and IgM showed that Nrg-1 was mainly effective subacutely. It is plausible that Nrg-1-mediated reduction in the spinal cord levels of IgM and IgG in subacute SCI reflects an indirect role for Nrg-1in modulation of vascular permeability. We have previously shown that Nrg-1 treatment attenuates the activity of matrix metalloproteinase (MMP)-9 in the injured spinal cord tissue [13]. MMP-9 is involved in the disruption of blood-spinal barrier [3]. Moreover, IL-6 can increase antibody production by B cells [82]. Thus, the Nrg-1-induced decline in IL-6 expression in our acute SCI studies can be another underlying cause for the reduced antibody deposition in the injured spinal cord at the subacute stage without alteration in B cell number. Our immunohistochemical analysis showed an increasing trend in IgG deposition in the spinal cord tissue with Nrg-1 treatment at chronic (42-day) time-point. This observation may reflect the modulatory effect of Nrg-1 on B cells that reside chronically in the spinal cord tissue. Our analysis of spinal cord B cells showed a close correlation between increased B_reg_ population in Nrg-1-treated animals and a trend towards higher production of IgG within the chronically injured spinal cord. This is in line with other studies that have attributed IgG production to B_reg_ cells [83]. Interestingly, IgG deposition by B_reg_ cells has been associated with beneficial immunomodulatory roles that include neutralizing harmful antigens from the microenvironment, inhibiting macrophages and dendritic cell activation, and enhancing the clearance of apoptotic bodies that contain self-antigens [83]. Moreover, studies by Nguyen and colleagues have also shown a positive role for IgG in recovery from SCI [84]. These studies revealed that systemic IgG administration increased IgG deposition in the injured spinal cord, which was associated with improved neural tissue preservation and functional recovery in a rat model of cervical SCI [84]. This evidence suggests that increased level of IgG may exert beneficial effects in SCI. Of note, our previous studies identified that Nrg-1 treatment improves tissue preservation in chronic SCI [13] that could be attributed, at least in part, to the increase in B_reg_ cells and IgG production.

## Conclusions

The present study, for the first time, implicates Nrg-1 as a positive regulator of neuroinflammation after SCI. We demonstrate that systemic bioavailability of Nrg-1 induces a pro-regenerative immune response in leukocytes that fosters a supportive environment for repair and regeneration in the injured spinal cord. Identification of a multifaceted immunoregulatory role for Nrg-1 establishes a novel therapeutic target for treating traumatic SCI and other CNS neuroinflammatory conditions.

## Additional file

Additional file 1: **Figure S1.** Flow cytometric verification of antibody specificity on injured and uninjured spinal cord tissue. (A–C) Spinal cord immune cells were gated for the detection of macrophages and their pro-inflammatory (M1, CD45^+^CD68^+^CD86^+^) and pro-regenerative (M2, CD45^+^CD68^+^CD163^+^) subpopulations. Our verification showed a negligible number of macrophages in the injured isotype control and no positively stained cells in the unstained control compared to the stained injured group confirming the specificity of antibodies used in our macrophage panel. **Figure S2.** Flow cytometric verification of antibody specificity for T cell detection. (A–C) Isolated spinal cord immune cells were stained and gated for the detection of helper T cells and their effector (T_eff_, CD3^+^CD4^+^IFNƔ^+^) and regulatory (T_reg_, CD3^+^CD4^+^IL-10^+^) subpopulations. A negligible number of T cells were detected in the injured isotype and the unstained control compared to the stained injured group confirming the specificity of antibodies used in our T cell panel. **Figure S3.** Specificity of the antibodies used for B cell detection was verified as shown above. (A–C) Isolated spinal cord immune cells were stained and gated for the detection of B cells and their (B_reg_, CD45RA^+^IL-10^+^) subpopulation. Our analysis showed a negligible number of B cells in the injured isotype control and no B cells in the unstained control group compared to stained injured group confirming the specificity of our B cell antibody panel. **Figure S4.** Immunohistochemical staining of the spinal cord sections at 1 mm caudal to the injury epicenter was performed to verify the tissue distribution of (A) macrophages/microglia (Iba-1^+^), (B) T cells (CD3^+^), and (C) B cells (CD45RA^+^) at 2 weeks post-injury. Dashed lines show the contour of the spinal cord section. Immune cells were mostly found within the SCI lesion. Magnified pictures and white arrows show the presence of (A) Iba-1^+^/IL-10^+^ macrophages/microglia, (B) CD3^+^/IL-10^+^ T cells, and (C) CD45RA^+^/L-10^+^ B cells, confirming the presence of these cells in the injured spinal cord tissue. (PDF 4416 kb)

## Abbreviations

aCSF: Artificial cerebrospinal fluid
ANOVA: Analysis of variance
B_reg_: Regulatory B cells
CCL: C-C motif ligands
CNS: Central nervous system
CSPGs: Chondroitin sulfate proteoglycans
CXCL: C-X-C motif ligands
CXCR: CXC chemokine receptor
DMEM: Dulbecco’s modified Eagle’s media
FoxP3: Forkhead box P3
HBSS: Hank’s Balanced Salt Solution
IL-10: Interleukin-10
IL-1β: Interleukin-1 beta
IL-6: Interleukin-6
MMP: Matrix metalloproteinase
NFkBIZ: NFκB inhibitor zeta
NF-κB: Nuclear factor kappa B
NO: Nitric oxide
Nrg-1: Neuregulin-1
PFA: Paraformaldehyde
RBC: Red blood cell
rhNrg-1: Recombinant human Nrg-1
SCI: Spinal cord injury
SD: Sprague-Dawley
SEM: Standard error of the mean
T_eff_: Effector T cells
TNF-⍺: Tumor necrosis factor-alpha
T_reg_: Regulatory T cells
