# Supplementary figures and images for: Neuregulin-1 elicits a regulatory immune response following traumatic spinal cord injury
Source: J Neuroinflammation. 2018 Feb 21;15:53. doi: 10.1186/s12974-018-1093-9 (PMC5822667; doi:10.1186/s12974-018-1093-9)

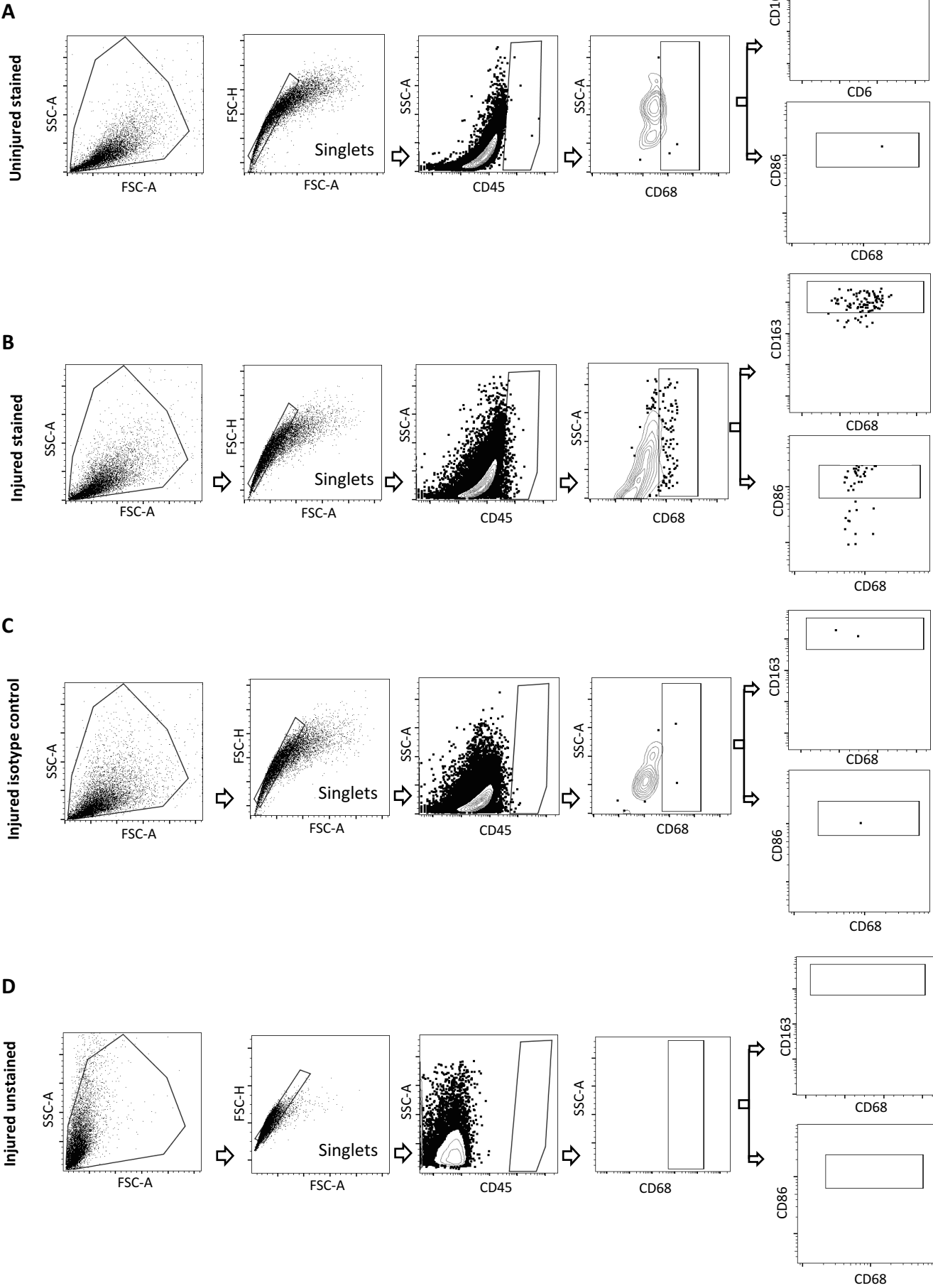

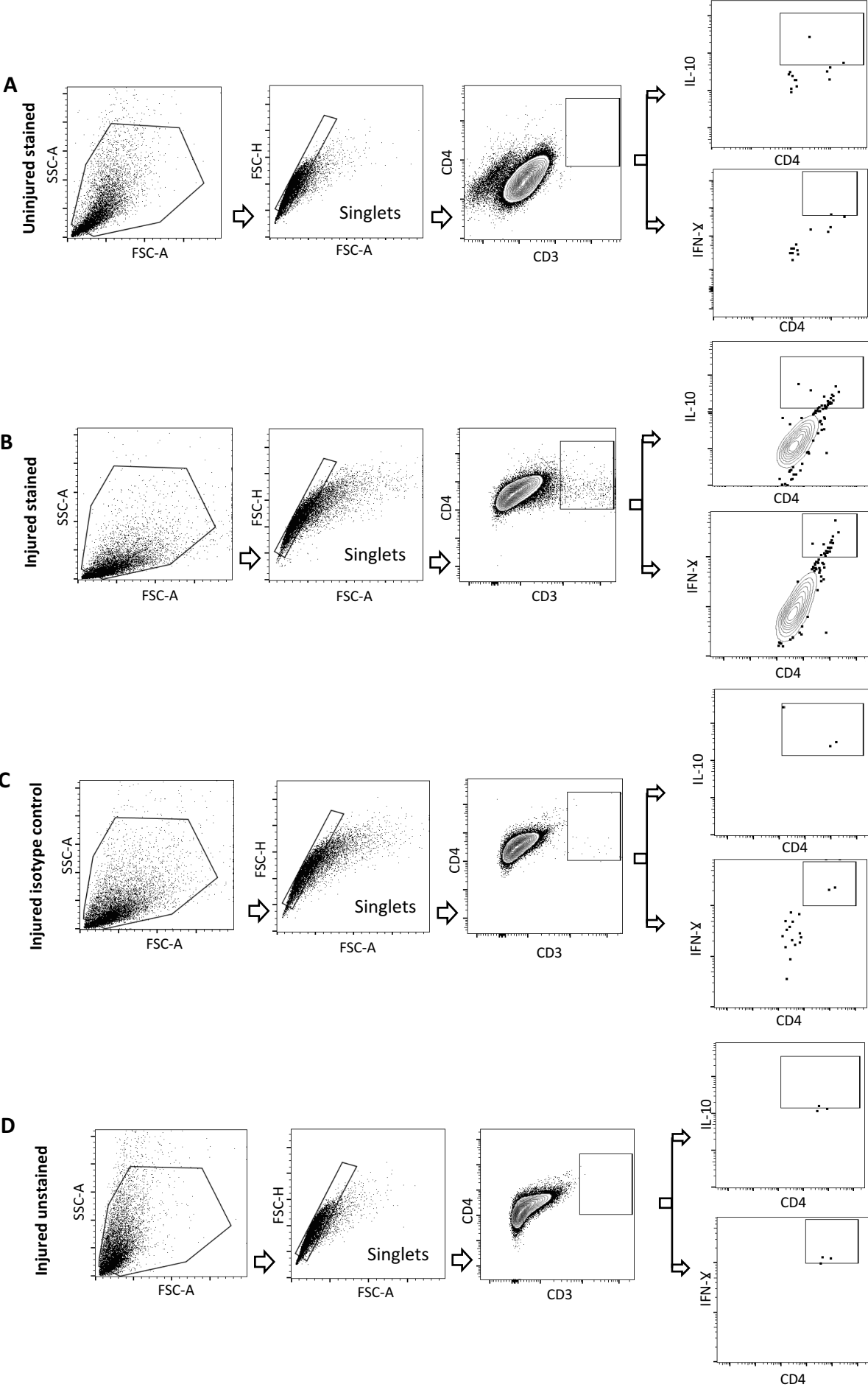

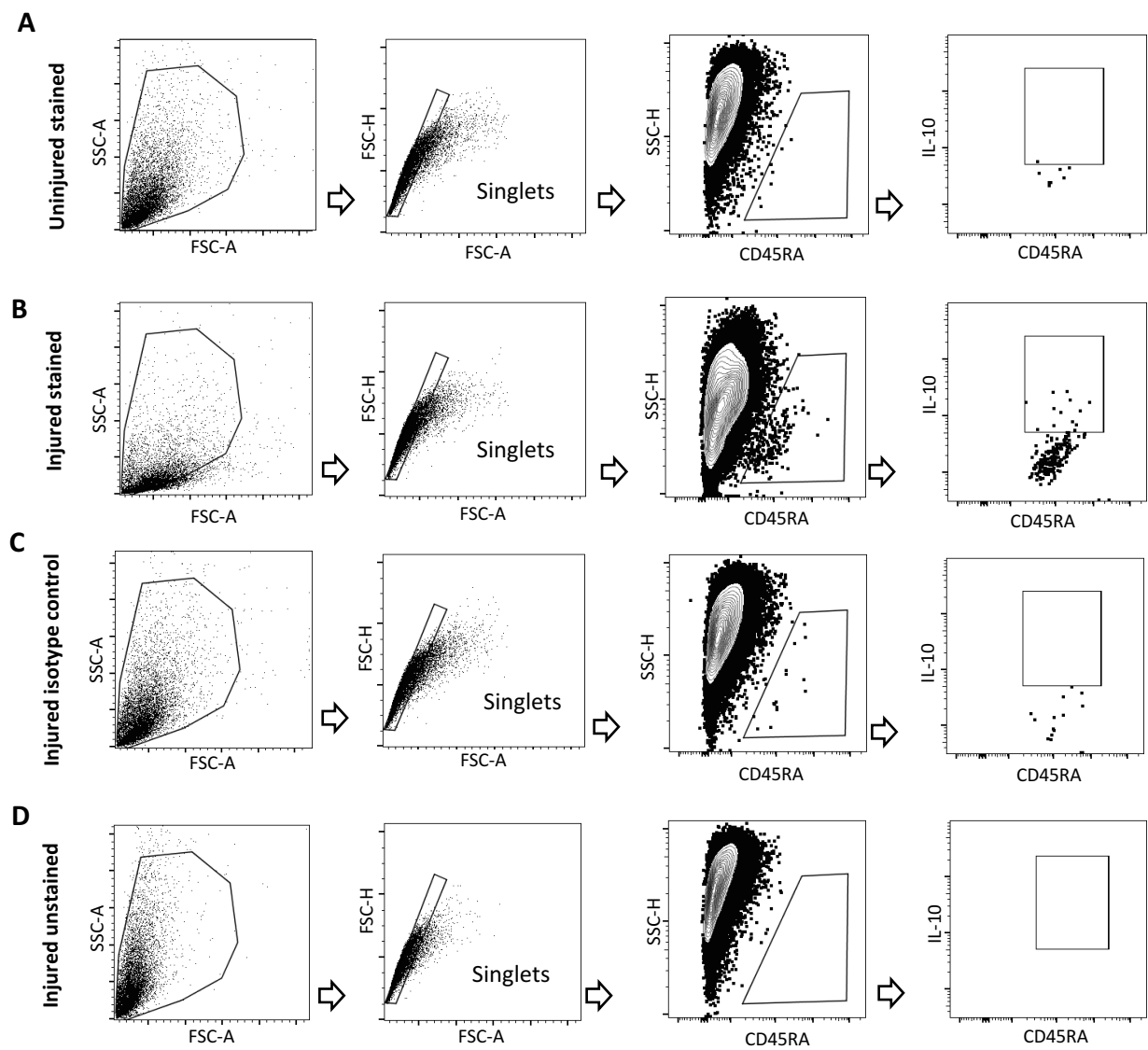

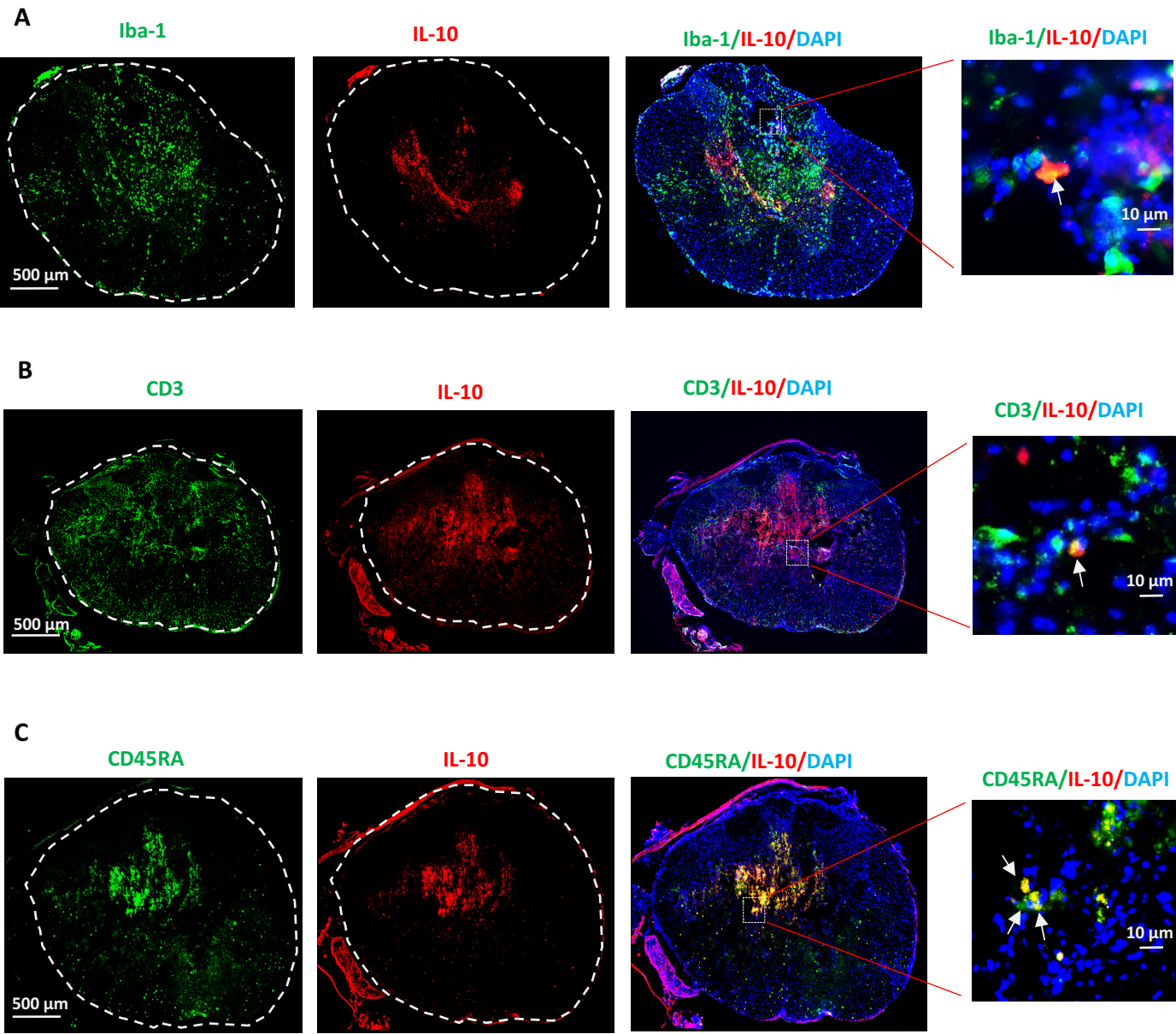

Supplement: Supplementary file 1 — Figure S1. Flow cytometric verification of antibody specificity on injured and uninjured spinal cord tissue. (A–C) Spinal cord immune cells were gated for the detection of macrophages and their pro-inflammatory (M1, CD45+CD68+CD86+) and pro-regenerative (M2, CD45+CD68+CD163+) subpopulations. Our verification showed a negligible number of macrophages in the injured isotype control and no positively stained cells in the unstained control compared to the stained injured group confirming the specificity of antibodies used in our macrophage panel. Figure S2. Flow cytometric verification of antibody specificity for T cell detection. (A–C) Isolated spinal cord immune cells were stained and gated for the detection of helper T cells and their effector (Teff, CD3+CD4+IFNƔ+) and regulatory (Treg, CD3+CD4+IL-10+) subpopulations. A negligible number of T cells were detected in the injured isotype and the unstained control compared to the stained injured group confirming the specificity of antibodies used in our T cell panel. Figure S3. Specificity of the antibodies used for B cell detection was verified as shown above. (A–C) Isolated spinal cord immune cells were stained and gated for the detection of B cells and their (Breg, CD45RA+IL-10+) subpopulation. Our analysis showed a negligible number of B cells in the injured isotype control and no B cells in the unstained control group compared to stained injured group confirming the specificity of our B cell antibody panel. Figure S4. Immunohistochemical staining of the spinal cord sections at 1 mm caudal to the injury epicenter was performed to verify the tissue distribution of (A) macrophages/microglia (Iba-1+), (B) T cells (CD3+), and (C) B cells (CD45RA+) at 2 weeks post-injury. Dashed lines show the contour of the spinal cord section. Immune cells were mostly found within the SCI lesion. Magnified pictures and white arrows show the presence of (A) Iba-1+/IL-10+ macrophages/microglia, (B) CD3+/IL-10+ T cells, and (C) CD45RA+/L-1 [file 12974_2018_1093_MOESM1_ESM.pdf]
